# Physicians’ Attitudes, Beliefs and Barriers to a Pulmonary Rehabilitation for COPD Patients in Saudi Arabia: A Cross-Sectional Study

**DOI:** 10.3390/healthcare10050904

**Published:** 2022-05-13

**Authors:** Abdulelah M. Aldhahir, Jaber S. Alqahtani, Saeed M. Alghamdi, Abdullah A. Alqarni, Shahad K. Khormi, Hassan Alwafi, Mohammed Samannodi, Rayan A. Siraj, Munyra Alhotye, Abdallah Y. Naser, Ali Hakamy

**Affiliations:** 1 Respiratory Therapy Department, Faculty of Applied Medical Sciences, Jazan University, Jazan 45142, Saudi Arabia; aohakamy@jazanu.edu.sa; 2 Department of Respiratory Care, Prince Sultan Military College of Health Sciences, Dammam 34313, Saudi Arabia; alqahtani-jaber@hotmail.com; 3 Respiratory Care Program, Faculty of Applied Medical Sciences, Umm Al-Qura University, Makkah 21961, Saudi Arabia; s.alghamdi18@imperial.ac.uk; 4 National Heart and Lung Institute, Imperial College, London SW7 2BX, UK; 5 Department of Respiratory Therapy, Faculty of Medical Rehabilitation Sciences, King Abdulaziz University, Jeddah 22254, Saudi Arabia; aaalqarni1@kau.edu.sa (A.A.A.); sha.1419@outlook.sa (S.K.K.); 6 Faculty of Medicine, Umm Al-Qura University, Mecca 21514, Saudi Arabia; hhwafi@uqu.edu.sa; 7 Department of Medicine, Faculty of Medicine, Umm Al-Qura University, Makkah 21514, Saudi Arabia; mssamannodi@uqu.edu.sa; 8 Department of Respiratory Therapy, College of Applied Medical Sciences, King Faisal University, Al-Hasa 31982, Saudi Arabia; rsiraj@kfu.edu.sa; 9 Department of Respiratory Therapy, King Saud Bin Abdulaziz University for Health Sciences, Riyadh 12271, Saudi Arabia; ma880@leicester.ac.uk; 10 Department of Applied Pharmaceutical Sciences and Clinical Pharmacy, Faculty of Pharmacy, Isra University, Amman 11622, Jordan; abdallah.naser@iu.edu.jo

**Keywords:** PR, COPD, pulmonary rehabilitation, Saudi Arabia, PR

## Abstract

This study aimed to assess physicians’ attitudes toward delivering pulmonary rehabilitation (PR) to chronic obstructive pulmonary disease (COPD) patients and identify factors and barriers that might influence referral decisions. Between September 2021 and January 2022, a cross-sectional online survey was distributed to all physicians in Saudi Arabia. A total of 502 physicians completed the online survey, of which 62.0% (*n* = 312) were male. General physicians accounted for 51.2%, while internal-medicine specialists and pulmonologists accounted for 26.9% and 6.6%, respectively. Only 146 (29%) physicians had referred COPD patients to a PR program. The difference in referral rates between all specialties (*p* = 0.011) was statistically significant. Physicians with more years of experience were more likely to refer COPD patients to PR (*p* < 0.001). Moreover, a home-based PR program was preferred by 379 physicians (75.5%), and 448 (89.2%) perceived smoking cessation as an essential component of PR. Availability of PR centers (69%) was the most common barrier for not referring patients to PR. The overall referral rate was low among all physicians, owing to a lack of PR centers and trained staff. Home-based delivery was the preferred method of delivering PR, with smoking cessation as an essential component.

## 1. Introduction

COPD is associated with poorly reversible airflow obstruction and is considered a common cause of morbidity and mortality worldwide, leading to a significant impact on overall health care costs [1]. Individuals with COPD tend to have daily symptoms of dyspnea, cough with or without sputum production, wheeze, and chest tightness. The worsening of these symptoms, referred to as ‘exacerbations’ in patients with COPD, is associated with reduced exercise capacity and increased susceptibility to frequent chest infections that might lead to hospital admissions [2].

Although COPD currently has no cure, the application of the available non-pharmacologic treatments can reduce the risk of exacerbation in patients with COPD. Among the non-pharmacologic interventions, PR serves as a core component of the management strategy for COPD [3]. PR is a comprehensive multidisciplinary intervention that provides exercise training and education to patients with chronic respiratory disease in order to improve quality of life and exercise performance [3,4]. The primary goals are to control and alleviate symptoms, improve activity tolerance, promote self-reliance and independence, decrease the need for acute resources, improve quality of life, improve treatment adherence, and prevent acute exacerbation [3,5]. Given that COPD is a multisystem manifestation that can produce significant systemic consequences (e.g., in the skeletal muscle, or cognitive effects), a multidisciplinary healthcare team should facilitate a PR program [3,5]. The supporting professional team varies globally and should include, but not be limited to, medical doctors, nurses, respiratory therapists, physiotherapists, psychologists, occupational therapists, dietitians, psychologists, and social workers. Among healthcare workers, medical doctors play an integral role in the implementation and administration of PR, given that referring COPD patients to PR programs is primarily carried out by physicians in Saudi Arabia [6].

Despite the existing evidence supporting the use of PR among the COPD population, PR services are not widely utilized in Saudi Arabia [7,8,9]. In addition, the perceptions of healthcare professionals, particularly physicians, of delivering PR programs in Saudi Arabia have not been explored before. To aid the implementation of PR in Saudi Arabia, it is important to identify the physicians’ attitudes and expectations regarding PR for patients with COPD. Therefore, this study aims to assess physicians’ attitudes and expectations toward delivering a PR program to COPD patients and to identify barriers and factors that might influence the decision of physicians to refer COPD patients to a PR program in Saudi Arabia.

## 2. Methods

### 2.1. Study Design

Between 15 September 2021, and 19 January 2022, a cross-sectional survey was conducted using an online platform (Survey Monkey).

### 2.2. Questionnaire Tool

The survey consisted of ten closed multiple-choice questions with free text spaces for extra comments; it was designed, created, and validated by respiratory medicine professionals based on the currently available literature [3,10,11]. After piloting the survey with ten healthcare experts with clinical experience in respiratory management, content and face validity were assessed prior to its initial dissemination.

The purpose of the study, as well as information on the lead investigator, were supplied to participants before they began answering the questionnaire. Furthermore, no personal information was collected; voluntary participation was ensured by asking if the participants were happy to complete the survey, and if they were, the survey was completed. The survey stated: “By answering yes in completing the survey question, you freely agree to engage in this study and offer your agreement to utilize your anonymous data for research purposes”. The survey took between three and five minutes to complete. The questionnaire consisted of two pages of structured responses divided into three sections, each with multiple-choice answers. The first section contained the respondents’ demographic information, including gender, profession, years of experience, responsibilities in the management of COPD, and whether the physicians had ever referred COPD patients to PR programs before. The second section consisted of three questions asking about the physicians’ perceptions of PR. The first question had six statements regarding the effectiveness of PR for COPD patients and used a 5-point Likert scale ranging from 1 “strongly disagree” to 5 “strongly agree”. The second question asked about additional components of PR aside from the exercise component, and the third question was about the ideal method of delivering PR to COPD patients. The third section included two questions regarding patient-related factors that influence referral decisions and process-related factors that influence the decision not to refer COPD patients. These questions used influence as a grading tool (no influence, some influence, and strong influence).

### 2.3. Study Population and Sampling Strategy

The study participants were recruited using convenience sampling techniques. The main target populations were general and specialized physicians, internal-medicine specialists, pulmonologists, and other physicians who worked with COPD patients or had prospective contact with this population. To reach a larger number of physicians working in Saudi Arabia, the survey was distributed through professional committees handling respiratory disorders, as well as social media (Twitter, WhatsApp, and Telegram). The study inclusion criteria were clearly stated in the invitation letter of the study.

### 2.4. Sample Size

Assuming a response distribution of 50%, the minimum sample size to conduct this research was 377, with a 5% margin of error and a 95% confidence level.

### 2.5. Ethical Approval

Institutional Review Board approval for the study was obtained from Jazan University, reference number (REC-43/03/040).

### 2.6. Statistical Analysis

Data were collected and analyzed using the Statistical Package for Social Sciences (SPSS software, Version 25). The categorical variables are reported and presented in percentages and frequencies. A chi-square (χ^2^) test was used to assess the statistically significant differences between categorical variables. Logistic regression was used to assess referral factors. Statistical significance was considered if *p* < 0.05.

## 3. Results

Between 15 September 2021, and 19 January 2022, 502 physicians (312 males (62%) and 190 females (38%)) completed the online survey. General physicians represented 51.2% of the respondents, followed by internal-medicine specialists (26.9%), pulmonologists (6.6%), and other specialties (15.3%) such as cardiology and thoracic surgery (Table 1). The majority of participants had three to six years of clinical experience with COPD patients; 23.7% had three to four years of experience and 22.1% had five to six years of experience (Table 1). The most prevalent responsibilities for the care for COPD patients were diagnosis (63.35%), admission prevention (63.35%), ongoing management (60%), and urgent assessment (58%) (Table 1).

### 3.1. Pulmonary Rehabilitation Referral Rate by Physicians’ Specialties

Out of 502 physicians, only 146 (29%) had referred COPD patients to a PR program. Surprisingly, around 73% of general physicians had never referred or were not sure if they had ever referred COPD patients to a PR program (Table 2). The pulmonologist group had a greater referral rate (54.5%) than the other groups. Overall, there was a statistically significant difference in referral rates across all disciplines (*p* = 0.011). When referral rates were compared between the general physicians and pulmonologists, pulmonologists were nearly four times more likely than general physicians to refer COPD patients to a PR program (OR: 3.8 (95% CI 1.7–8.7); *p* < 0.001).

When all physicians, disregarding specialty, with less than a year of experience, were compared with other groups of clinical experience, physicians with one to two years of clinical experience with COPD patients were three times more likely to refer COPD patients to a PR program (OR: 3.4 (95% CI 1–11.3): *p* = 0.04). Physicians with three to four years of clinical experience with COPD patients were almost four times more likely to refer COPD patients to a PR program (OR: 3.7 (95% CI 1.2–11.44); *p* = 0.02). Physicians with five to six years of clinical experience with COPD patients were almost six times more likely to refer COPD patients to a PR program (OR: 5.7 (95% CI 1.8–17.3); *p* = 0.002). Physicians with seven to eight years of clinical experience with COPD patients were six times more likely to refer COPD patients to a PR program (OR: 6.3 (95% CI 1.9–20.2); *p* = 0.002). Physicians with nine to ten years of clinical experience with COPD patients were almost eleven times more likely to refer COPD patients to a PR program (OR: 10.8 (95% CI 2.97–39.12); *p* < 0.000). Physicians with more than ten years of experience were eight times more likely to refer COPD patients to a PR program (OR: 8.3 (95% CI 2.6–26.4); *p* < 0.000). Overall, COPD patients were more likely to be referred to PR by physicians with more years of experience (*p* < 0.001).

### 3.2. Physicians’ Opinions on Referring COPD Patients, Mode of Delivery and Component of Pulmonary Rehabilitation

A total of 313 (62%) out of 502 physicians strongly agreed that PR would improve patients’ exercise capacity, while 136 (27%) agreed. Furthermore, 227 (55%) strongly agreed and 157 (31%) agreed that PR would alleviate dyspnea and fatigue in patients with COPD (Table 3). The majority of physicians agreed that PR would improve COPD patients’ health-related quality of life, with 281 (56%) strongly agreed and 174 (34.7%) agreed. A total of 259 physicians (51.6%) strongly agreed and 163 (32.5%) agreed that PR would reduce the risk of future COPD exacerbation, whereas 269 (53.6%) strongly agreed and 167 (33.3%) agreed that PR would improve patients’ disease self-management. A total of 379 (75.5%) of the 502 physicians thought that delivering a PR program at home is the best option, followed by 321 (63.9%) who favored in-hospital supervised programs. In contrast, a tailored program with healthcare provider support on the telephone was the least chosen method of delivering a PR program, 112 respondents (22.3%). Aside from the exercise component, 448 (89.2%), 406 (80.9%), and 402 (80.1%) physicians deemed smoking cessation, symptom management, and education about COPD disease to be essential components of PR, respectively (Table 3).

### 3.3. Patient-Related Factors That Influence Referral Decision to Pulmonary Rehabilitation

**Figure 1:**
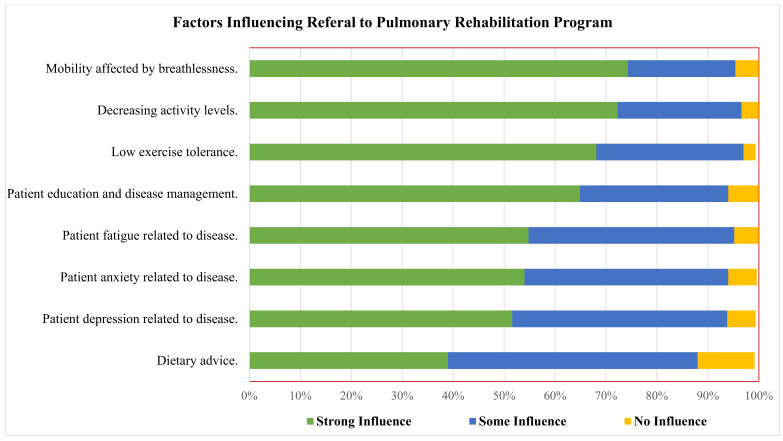
Patient-related factors that influence referral decision to PR, using strong, some or no influence grading.

Mobility impacted by breathlessness (74.0%), decreased activity levels (72.0%), low exercise tolerance (68.0%), and patients’ education and illness management (65.0%) were the patient-related factors that greatly influenced physician referral decisions to PR (Figure 1).

### 3.4. Pulmonary Rehabilitation Referral Barriers

**Figure 2:**
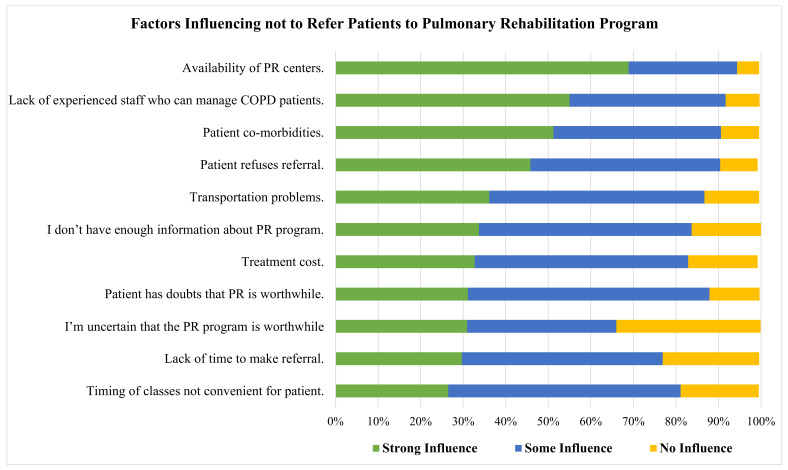
Barriers for referring COPD patients to PR, using strong, some or no influence grading.

The availability of PR facilities (69.0%), lack of experienced personnel who can handle COPD patients (55.0%), patient co-morbidities (51.0%), and patients refusing referral (46.0%) were the process-related variables that strongly impacted physicians to not refer COPD patients to PR (Figure 2).

## 4. Discussion

To the best of our knowledge, this is the first study to investigate a range of physicians’ perceptions of PR program benefits and experiences of referring or considering a referral to PR programs for patients with COPD in Saudi Arabia. In this nationwide survey, physicians agreed overall on the benefits of PR, irrespective of their specialties. Although pulmonologists had higher referral rates compared with other specialties, the overall referral rates to PR programs were significantly lower, demonstrating a clear gap in the current practice. This was mainly attributed to the lack of PR centers and a shortage of trained staff who could manage patients with COPD. Home-based PR was perceived as the most suitable way of delivering a PR program, and smoking cessation was the most essential component of PR aside from exercise capacity.

PR is increasingly recognized as the most effective non-pharmacological intervention for patients with COPD. It alleviates dyspnea, ameliorates depressive symptoms, improves functional and exercise capacity, reduces hospital admissions, and improves survival [3,5,12]. Although this study demonstrated a consensus about these benefits among physicians, referral rates were significantly low at 29%. While this may be unexpected, the existing literature generally agrees on the suboptimal referral rate. A previous systematic review showed that 93% of the included observational studies (*n* = 26/28) reported referral rates of less than 35% [13], in line with the findings of our study. Although the methods used to measure referrals may not have been sufficient, in recent years, no efforts have been made to improve referral rates. Assuming the latter is true, a targeted intervention to overcome any barriers and, consequently, improve referrals is needed. A proposed way to enhance referrals is to target the physicians responsible for carrying out PR referrals.

Referral rates varied among physicians’ specialties, with pulmonologists having higher rates of referral compared with other specialties. We demonstrated that pulmonologists are almost four times more likely to refer patients to PR compared with GPs, in line with a study conducted by Tang and colleagues, which found that patients who were managed by a respiratory team were more likely (OR: 2.6) to receive PR referrals compared with those managed by GPs [14]. Reasons for this could be that physicians from other specialties may be unfamiliar with the availability of PR centers or existing referral guidelines, despite their availability within the healthcare system. Pulmonologists are more likely to have more training and experience with PR and are routinely involved in the provision of PR programs, compared with other specialties [15], hence contributing to higher referral rates. In addition, physicians with more years of experience were more likely to refer patients to PR programs, which may indicate better knowledge of the benefits of PR, greater familiarity with local resources, and higher exposure to COPD cases. Providing more education (e.g., courses on COPD management and COPD seminars) to junior physicians and other healthcare professionals to increase PR referrals is therefore vital.

One of the most frequently mentioned barriers to PR referral in this study was the availability of PR centers. In Saudi Arabia, a limited number of PR programs are available, which could serve only a limited number of patients with COPD [7]. This identifies a considerable gap in the practice and highlights the need to establish new programs that meet the international criteria across the country. However, PR can be provided within the existing infrastructure and using workers in the hospitals [16]. A previous study demonstrated that an outpatient PR program offered at a small hospital was as effective as PR provided by a large hospital [17]. Other models in which a PR program can be offered include those offered to inpatients or those that are community-based [17,18].

Another major barrier to PR referral reported in this study is the lack of well-trained staff. Saudi Arabia suffers from a shortage of healthcare workers, which may affect the quality of COPD care [19]. Previous reports state that the number of chest physicians in Saudi Arabia is low, and the number of specialized nurses is even lower [19,20]. Moreover, only a limited number of disciplines manage patients with COPD, although evidence suggests that COPD management by a multidisciplinary team is much better [19,21]. Lack of awareness regarding the management of patients with COPD and/or lack of knowledge regarding the multidisciplinary approach may explain the lack of specialized healthcare workers [22]. The government should provide training incentives to upskill current healthcare staff. In addition, offering high-quality education in Saudi Arabia or sending students to international universities to specialize in respiratory medicine can be useful in stimulating this change.

The finding that a home-based setting was perceived as the most suitable way to deliver a PR program may partially be explained by the limited number of PR centers in the country [7]. The COVID-19 pandemic, during which a curfew was imposed and most face-to-face PR services were forced to close temporarily, may have contributed to this. In the case of Saudi Arabia, a home-based setting seems to be a viable option to deliver an effective PR program, given the lack of manpower and available PR centers. Home-based PR is as effective as a conventional PR program in improving dyspnea and exercise capacity [23,24]. Taken together, hospitals may utilize their available resources, even if they are limited, to deliver an effective PR program using any of the models, one of which is a home-based setting [25].

In this study, smoking cessation was perceived as the most essential component of PR programs aside from exercise capacity, in line with the current AT/ERS official consensus on the essential components of PR [10,11]. Smoking remains the leading cause of COPD. Additionally, the prevalence of smoking among diagnosed COPD patients ranges from 38% to 77% and is responsible for the deaths of 73% of COPD patients worldwide [26,27]. Furthermore, smoking is responsible for accelerating lung function decline, worsening symptoms, and higher exacerbations [28,29]. Moreover, the number of cigarettes smoked by individuals with COPD was significantly lower after joining a PR program [30]. However, smoking remains responsible for a higher PR dropout rate among COPD patients [11,30]. Therefore, smoking cessation is an essential component of successful PR and should be offered to COPD patients who currently smoke.

### Limitations

Although we have reached the a priori desired level of power in this study, a convenience sample strategy was used, which might provide a potential selection bias. The number of respiratory physicians was very low, which was due to their limited number in the Kingdom of Saudi Arabia. Our study did not survey or interview other healthcare providers who were part of COPD management. Last, this study was conducted during the COVID-19 pandemic, which might affect physicians’ opinions, especially concerning the mode of PR delivery.

## 5. Conclusions

PR benefits are well-recognized among physicians irrespective of their specialties. However, the overall referral rates to PR programs were significantly lower, except for referrals by pulmonologists. This was mainly attributed to the lack of PR centers and a shortage of trained staff. Consequently, home-based PR was the most suitable way of delivering PR, while smoking cessation was the most essential component of the PR program, aside from the exercise component. Further studies are needed to assess non-physicians’ attitudes and expectations toward delivering a PR program to COPD patients and to identify barriers and factors that might influence the referral decisions in Saudi Arabia.

## Figures and Tables

**Table 1:** Demographic data and characteristics of all study respondents (*n* = 502).

Demographic Variables	Frequency (%)
*Gender*	
Male Female	312 (62.0%) 190 (38.0%)
*Profession*	
Pulmonologist Internal medicine General physician Other	33 (6.6%) 135 (26.9%) 257 (51.2%) 77 (15.3%)
*Years of experience with COPD patients*	
<1 year 1–2 years 3–4 years 5–6 years 7–8 years 9–10 years >10 years	46 (9.2%) 64 (12.7%) 119 (23.7%) 111 (22.1%) 68 (13.5%) 29 (5.8%) 65 (12.9%)
*Responsibilities for the care of COPD patients*	
Diagnosis Urgent assessments Non-urgent care Ongoing management Admission prevention Medication check Prescribing Oxygen therapy In-patient treatment Outpatient clinics Primary care Other	318 (63.3%) 291 (58.0%) 252 (50.2%) 301 (60.0%) 318 (63.3%) 225 (44.8%) 240 (47.8%) 206 (41.0%) 231 (46.0%) 238 (47.4%) 194 (38.6%) 13 (2.6%)

Data are presented as frequencies and percentages. Abbreviations: COPD, chronic obstructive pulmonary disease.

**Table 2:** Patient referral rate per physician specialty (*n* = 502).

Item	Frequency (%)
Patient Referral Pulmonologist	
Yes No Not sure	18 (54.5%) 10 (30.0%) 5 (15.0%)
*Internal medicine*	
Yes No Not sure	42 (31.0%) 71 (52.5%) 22 (16.2%)
*General physician*	
Yes No Not sure	70 (27.2%) 148 (57.5%) 39 (15.1%)
*Other profession*	
Yes No Not sure	16 (20.8%) 52 (67.5%) 9 (11.6%)

Data are presented as frequencies and percentages.

**Table 3:** Physicians’ perceptions of referring COPD patients to PR, mode of delivery and component of PR (*n* = 502).

Item	Frequency (%)
**Physicians’ perception of referring COPD patients to PR**	
*I believe PR will improve patients’ exercise capacity*	
Strongly agree Agree Neutral Disagree Strongly disagree	313 (62.2%) 136 (27.1%) 22 (4.4%) 4 (0.8%) 27 (5.4%)
*I believe PR would reduce dyspnea and fatigue*	
Strongly agree Agree Neutral Disagree Strongly disagree	227 (55.2%) 157 (31.3%) 39 (7.8%) 8 (1.6%) 21 (4.2%)
*I believe PR will improve patients’ anxiety and depression*	
Strongly agree Agree Neutral Disagree Strongly disagree	231 (46.0%) 187 (37.3%) 58 (11.6%) 6 (1.2%) 20 (4%)
*I believe PR will improve patients’ health-related quality of life*	
Strongly agree Agree Neutral Disagree Strongly disagree	281 (56.0%) 174 (34.7%) 20 (4.0%) 8 (1.6%) 19 (3.8%)
*I believe PR will reduce the risk of future COPD exacerbation*	
Strongly agree Agree Neutral Disagree Strongly disagree	259 (51.6%) 163 (32.5%) 47 (9.4%) 14 (2.8%) 19 (3.8%)
*I believe PR will reduce hospital readmission*	
Strongly agree Agree Neutral Disagree Strongly disagree	262 (52.2%) 164 (32.7%) 48 (9.6%) 12 (2.4%) 16 (3.2%)
*I believe PR will improve patients’ nutritional status*	
Strongly agree Agree Neutral Disagree Strongly disagree	217 (43.2%) 180 (35.9%) 75 (14.9%) 13 (2.6%) 17 (3.4%)
*I believe PR will improve patients’ disease self-management*	
Strongly agree Agree Neutral Disagree Strongly disagree	269 (53.6%) 167 (33.3%) 36 (7.2%) 10 (2.0%) 20 (4.0%)
**The best way to deliver a PR program for COPD patients**	
At home. In-hospital supervised program. Online program with healthcare provider support. Tailored program with healthcare provider support through phone.	379 (75.5%) 321 (63.9%) 223 (44.4%) 112 (22.3%)
**Component of PR program aside from exercise component**	
Smoking cessation Symptoms management Information about COPD disease Psychological support Information about medications Nutritional counseling	448 (89.2%) 406 (80.9%) 402 (80.1%) 363 (72.3%) 318 (63.3%) 267 (53.2%)

Data are presented as frequencies and percentages. Abbreviations: COPD, chronic obstructive pulmonary disease; PR, pulmonary rehabilitation.

## Data Availability

Supporting data are available on request.

## References

[1] Mathers C.D., Loncar D. (2006). Projections of global mortality and burden of disease from 2002 to 2030. PLoS Med..

[2] Alqahtani J.S., Njoku C.M., Bereznicki B., Wimmer B.C., Peterson G.M., Kinsman L., Aldabayan Y.S., Alrajeh A.M., Aldhahir A.M., Mandal S. (2020). Risk factors for all-cause hospital readmission following exacerbation of COPD: A systematic review and meta-analysis. Eur. Respir. Rev..

[3] Spruit M.A., Singh S.J., Garvey C., ZuWallack R., Nici L., Rochester C., Hill K., Holland A.E., Lareau S.C., Man W.D.C. (2013). An official American Thoracic Society/European Respiratory Society statement: Key concepts and advances in pulmonary rehabilitation. Am. J. Respir. Crit. Care Med..

[4] Evans R.A., Singh S.J. (2019). Minimum important difference of the incremental shuttle walk test distance in patients with COPD. Thorax.

[5] Vogelmeier C.F., Criner G.J., Martinez F.J., Anzueto A., Barnes P.J., Bourbeau J., Celli B.R., Chen R., Decramer M., Fabbri L.M. (2017). Global strategy for the diagnosis, management, and prevention of chronic obstructive lung disease 2017 report. Am. J. Respir. Crit. Care Med..

[6] Sakkijha H., Idrees M.M. (2014). Saudi Guidelines on the Diagnosis and Treatment of Pulmonary Hypertension: Pulmonary hypertension due to lung diseases and/or hypoxia. Ann. Thorac. Med..

[7] Aldhahir A.M., Alghamdi S.M., Alqahtani J.S., Alqahtani K.A., Al Rajah A.M., Alkhathlan B.S., Singh S.J., Mandal S., Hurst J.R. (2021). Pulmonary rehabilitation for COPD: A narrative review and call for further implementation in Saudi Arabia. Ann. Thorac. Med..

[8] Al Moamary M.S., Tamim H.M., Al-Mutairi S.S., Al-Khouzaie T.H., Mahboub B.H., Al-Jawder S.E., Alamoudi O.S., Al Ghobain M.O. (2012). Quality of life of patients with chronic obstructive pulmonary disease in the Gulf Cooperation Council countries. Saudi Med. J..

[9] Al-Moamary M.S., Köktūrk N., Idrees M.M., Şen E., Juvelekian G., Abi Saleh W., Zoumot Z., Behbehani N., Hatem A., Masoud H.H. (2021). Unmet need in the management of chronic obstructive pulmonary disease in the Middle East and Africa region: An expert panel consensus. Respir. Med..

[10] Hill K., Vogiatzis I., Burtin C. (2013). The importance of components of pulmonary rehabilitation, other than exercise training, in COPD. Eur. Respir. Rev..

[11] Holland A.E., Cox N.S., Houchen-Wolloff L., Rochester C.L., Garvey C., ZuWallack R., Nici L., Limberg T., Lareau S.C., Yawn B.P. (2021). Defining Modern Pulmonary Rehabilitation. An Official American Thoracic Society Workshop Report. Ann. Am. Thorac. Soc..

[12] Alqahtani J.S., Alghamdi S.M., Aldhahir A.M., Althobiani M., Oyelade T. (2021). Key toolkits of non-pharmacological management in COPD: During and beyond COVID-19. Front. Biosci..

[13] Milner S.C., Boruff J.T., Beaurepaire C., Ahmed S., Janaudis-Ferreira T. (2018). Rate of, and barriers and enablers to, pulmonary rehabilitation referral in COPD: A systematic scoping review. Respir. Med..

[14] Tang C.Y., Taylor N.F., McDonald C.F., Blackstock F.C. (2014). Level of adherence to the GOLD strategy document for management of patients admitted to hospital with an acute exacerbation of COPD. Respirology.

[15] Perez X., Wisnivesky J.P., Lurslurchachai L., Kleinman L.C., Kronish I.M. (2012). Barriers to adherence to COPD guidelines among primary care providers. Respir. Med..

[16] Jenkins S., Hill K., Cecins N.M. (2010). State of the art: How to set up a pulmonary rehabilitation program. Respirology.

[17] Ward J.A., Akers G., Ward D.G., Pinnuck M., Williams S., Trott J., Halpin D.M. (2002). Feasibility and effectiveness of a pulmonary rehabilitation programme in a community hospital setting. Br. J. Gen. Pract..

[18] Maltais F., Bourbeau J., Shapiro S., Lacasse Y., Perrault H., Baltzan M., Hernandez P., Rouleau M., Julien M., Parenteau S. (2008). Effects of home-based pulmonary rehabilitation in patients with chronic obstructive pulmonary disease: A randomized trial. Ann. Intern. Med..

[19] Alsubaiei M., Cafarella P., Frith P., McEvoy R., Effing T. (2018). Factors influencing management of chronic respiratory diseases in general and chronic obstructive pulmonary disease in particular in Saudi Arabia: An overview. Ann. Thorac. Med..

[20] Aboshaiqah A. (2016). Strategies to address the nursing shortage in Saudi Arabia. Int. Nurs. Rev..

[21] Kuzma A.M., Meli Y., Meldrum C., Jellen P., Butler-Lebair M., Koczen-Doyle D., Rising P. (2008). Multidisciplinary care of the patient with chronic obstructive pulmonary disease. Proc. Am. Thorac. Soc..

[22] Alsubaiei M.E., Frith P.A., Cafarella P.A., Quinn S., Al Moamary M.S., McEvoy R.D., Effing T.W. (2017). COPD care in Saudi Arabia: Physicians’ awareness and knowledge of guidelines and barriers to implementation. Int. J. Tuberc. Lung Dis..

[23] Horton E.J., Mitchell K.E., Johnson-Warrington V., Apps L.D., Sewell L., Morgan M., Taylor R.S., Singh S.J. (2018). Comparison of a structured home-based rehabilitation programme with conventional supervised pulmonary rehabilitation: A randomised non-inferiority trial. Thorax.

[24] Alghamdi S.M., Rajah A.M.A., Aldabayan Y.S., Aldhahir A.M., Alqahtani J.S., Alzahrani A.A. (2021). Chronic Obstructive Pulmonary Disease Patients’ Acceptance in E-Health Clinical Trials. Int. J. Environ. Res. Public Health.

[25] McCarthy B., Casey D., Devane D., Murphy K., Murphy E., Lacasse Y. (2015). Pulmonary rehabilitation for chronic obstructive pulmonary disease. Cochrane Database Syst. Rev..

[26] MacNee W. (2010). Chronic Obstructive Pulmonary Disease.

[27] Tønnesen P. (2013). Smoking cessation and COPD. Eur. Respir. Rev..

[28] Badaran E., Ortega E., Bujalance C., Del Puerto L., Torres M., Riesco J.A. (2012). Smoking and COPD exacerbations. Eur. Respir. J..

[29] Alqahtani J.S., Aldhahir A.M., Oyelade T., Alghamdi S.M., Almamary A.S. (2021). Smoking cessation during COVID-19: The top to-do list. Npj Prim. Care Respir. Med..

[30] Brown A.T., Hitchcock J., Schumann C., Wells J.M., Dransfield M.T., Bhatt S.P. (2016). Determinants of successful completion of pulmonary rehabilitation in COPD. Int. J. Chronic Obstr. Pulm. Dis..

